# Efficacy of Alternative Cardiac Rehabilitation Delivery Formats in Improving Psychological Symptoms after Coronary Artery Bypass Grafting 

**Published:** 2018-07

**Authors:** Mozhgan Saeidi, Ali Soroush, Saeid Komasi, Agostino Brugnera, Marco Patucelli, Danilo Carrozzino, Mario Fulcheri, Angelo Compare

**Affiliations:** 1 *Cardiac Rehabilitation Center, Imam Ali Hospital, Kermanshah University of Medical Sciences, Kermanshah, Iran.*; 2 *Heart Research Center, Imam Ali Hospital, Kermanshah University of Medical Sciences, Kermanshah, Iran.*; 3 *Clinical Research Development Center, Imam Reza Hospital, Kermanshah University of Medical Sciences, Kermanshah, Iran.*; 4 *Department of Human and Social Sciences, University of Bergamo, Bergamo, Italy.*; 5 *University “G. d'Annunzio” of Chieti and Pescara, Chieti, Italy.*

**Keywords:** *Coronary artery bypass*, *Cardiovascular rehabilitation*, *Hospital centralized services*, *Home care services*

## Abstract

**Background:** Health-care systems always strive for alternative cardiac rehabilitation (CR) models to ensure that options available to patients better fit their needs, risk factor profiles, and preferences. We assessed the effects of hospital-based cardiac rehabilitation (HBCR) and hybrid cardiac rehabilitation (HCR) on psychological symptoms (i.e., anxiety, depression, and stress) among patients following coronary artery bypass graft surgery (CABG).

**Methods:** This cross-sectional study recruited 115 CABG patients who were referred to the Department of Cardiac Rehabilitation at Imam Ali Hospital in the Iranian city of Kermanshah between January and May 2016. The patients were assigned to 1 of the 2 programs of HBCR (26 sessions) and HCR (10 sessions). The study population’s psychological symptoms were assessed using the Depression, Anxiety, and Stress Scale (DASS) before and after the intervention, and the data were analyzed using paired t-tests and ANCOVA.

**Results:** A total of 105 (91%) patients concluded the CR program. Respectively, 50.8% and 26.2% of the participants in the HBCR and HCR programs were female. The mean age was 59.6±9.2 years in the HBCR group and 58.7±6.1 years in the HCR group. The ANCOVA results indicated that both HBCR and HCR programs were equally effective in reducing anxiety (P=0.001 vs. P=0.015) and stress (P=0.002 vs. P=0.003) among the CABG patients, while only HBCR was effective in alleviating depressive symptoms (P=0.001).

**Conclusion:** Our results demonstrated the efficacy of HBCR and HCR in diminishing stress and anxiety levels among the CABG patients. However, depression was affected only by HBCR.

## Introduction

Outpatient cardiac rehabilitation (CR) is one of the most important methods in the control and reduction of consequent complications of invasive cardiac treatments.^1^^, ^2 Past studies have indicated that CR has a significant role in reducing mortality.^1^^, ^^2^ CR is delivered in different formats. The oldest one is hospital-based cardiac rehabilitation (HBCR), which has been recently criticized due to its cost and relative unavailability.^   3 ^ Therefore, health-care systems always seek alternative models in order to ensure that options afforded to patients are better compatible with their needs, risk factor profiles, and preferences.^        4 ^ New interventions include multi-factorial individualized call-service, internet-based delivery, call-services focused on exercise or recovery, home-based CR, rural area programs, and remote populations within different cultures and languages.^4^^, ^^5^ 

Hybrid cardiac rehabilitation (HCR) is one of the newest programs; it is effective in controlling cardiac risk factors, ^3^^, ^^4^^, ^^6^ promoting exercise capacity, and increasing the commitment to treatment.^7^ In addition, economic evaluations have shown that HCR programs seem to cost 62% less than HBCR ones.^8^ The efficacy of HCR in reducing the consequences of disease and promoting healthy behaviors in patients is well-known.^3^^, ^^4^^, ^^6^^-^^9^ However, there have been few studies on the effects of this program on psychological symptoms after invasive cardiac interventions. Therefore, the present study aimed to assess and compare the effects of HCR and HBCR on psychological symptoms (i.e., anxiety, depression, and stress) among cardiac patients. 

## Methods

In this cross-sectional study, a number of cardiovascular patients admitted to Imam Ali Hospital (Kermanshah, Iran) for coronary artery bypass graft surgery (CABG) volunteered to participate before hospital discharge. Kermanshah city is the seat of the homonymous province in the western part of Iran and is situated 326 miles away from Tehran (capital of Iran). Based on the census of 2011, the population of this city was 851 405 persons. Imam Ali Hospital of Kermanshah is a government hospital with a capacity of 214 beds.^10^

Patients meeting the present study’s inclusion criteria were identified by the members of the CR team and were asked to participate in this study. The study was conducted in accordance with the guidelines of the Ethics Committee of Kermanshah University of Medical Sciences and the Declaration of Helsinki. After obtaining informed consent from the participants, the researchers reassured the patients about the confidentiality of the data collected. The patients’ data were collected at the beginning and at the end of both programs.

The inclusion criteria were comprised of age between 30 and 80 years, being able to understand and speak Farsi fluently, not using psychiatric medications, and having no physical limitations that would preclude participation in exercising programs. Patients were excluded if they had severe chest pain or persistent dyspnea during aerobic exercise and if they participated in fewer than 90% of the CR sessions.^   2 ^

The Depression, Anxiety, and Stress Scale (DASS) is a 21-item questionnaire that evaluates the 3 dimensions of depression, anxiety, and stress (7 items for each subscale). Each item is rated on a 4-point Likert scale (i.e., never, few, many, or too many). Previous studies have reported a Cronbach’s alpha of 0.81, 0.73, and 0.81, respectively, for the depression, anxiety, and stress subscales.^11^ The Cronbach’s alpha in the Iranian population was 0.70, 0.66, and 0.76, correspondingly, for the depression, anxiety, and stress subscales. Moreover, the convergent validity of the test has been previously approved.^12^ In addition, the Cronbach’s alpha in the current study was 0.67, 0.63, and 0.80 for the depression, anxiety, and stress subscales, respectively. In the present study, the DASS questionnaire was administered at the onset of CR and a week after the completion of the course.

The patients were divided into 2 programs. The high-risk patients were assigned to the HBCR program after a cardiology-based interview in order for them to exercise in a perfectly controlled situation, and the patients with a low-medium risk who both provided consent and resided far from the hospital were assigned to the HCR program. The HBCR patients participated in a 1-hour exercise session that consisted of warm-up for 10 minutes, dynamic exercise for 45 minutes, and recovery for 5 minutes, 3 times a week, totaling 26 sessions over 8 weeks. In comparison, the HCR patients participated in 2-hour training sessions and a 1-hour exercise session (10 weekly sessions). The training sessions focused on the management of risk factors, healthy nutrition, weight control, and stress management. A family member was asked to participate in the training sessions if the patient was illiterate. At the end of every session, the patients were asked to do aerobic exercises such as walking up to 5 days of a week and performing relaxation techniques. The patients themselves recorded the results. Additionally, the patients were asked to call a member of the CR team during the week to provide an update and answer some questions.^7^

The HBCR and HCR groups were compared using the χ^2 ^test for the nominal and categorical variables (i.e., gender, education level, occupation, smoking, and histories related to myocardial infarction, hypertension, diabetes, and hyperlipidemia) and the independent sample *t*-tests for the continuous variables (i.e., age, depression, anxiety, and stress). Additionally, the percentages of the noncontinuous variables and the means and standard deviations of the continuous variables were reported. Finally, a paired sample *t*-test was applied to assess the effects of each program on psychological symptoms. The efficacy of the 2 programs was assessed using ANCOVA after statistical assumptions were checked. In all the analyses, depression, anxiety, and stress were the dependent variables and the delivery formats including HBCR and HCR were the independent variables. The effects of the confounding variables including all the demographic factors (i.e., age, gender, education level, and occupation) and the covariates including psychological symptoms at baseline were controlled using ANCOVA. All the statistical analyses were performed utilizing the statistical software SPSS, version 21.0 (Armonk, NY: IBM Corp). All the statistical tests were 2-sided, and a p value equal to or less than 0.05 was considered significant. 

## Results

Among the 123 CABG patients admitted to the program of CR between January and May 2016, a total of 115 patients (105 patients in the final assessment) met the inclusion criteria. The patients were referred for CR within 30 to 60 days after surgery. The demographic data and the baseline psychological symptoms of the study population are depicted in Table 1, which demonstrates no significant differences between the 2 groups with the exception of the variables of gender and occupation.

Table 2 shows the effects of each CR programs on psychological symptoms. Based on the results, HBCR was effective in reducing depression (P<0.001), anxiety (P<0.001), and stress (P=0.002). In contrast, HCR was effective only in alleviating anxiety levels (P=0.015) and stress (P=0.003). Table 3 illustrates the differences in terms of psychological symptoms between the 2 groups. After controlling was done for the demographic variables (i.e., age, gender, education level, and occupation) and the covariates (dependent variables at baseline), the ANCOVA indicated that both programs were effective in reducing anxiety and stress levels. 

**Table 1:** Baseline demographics and psychological symptoms of the study population (N=105)*

	HBCR (n=63)	HCR (n=42)	P
Age (y)	59.659.18	58.716.15	0.533
Sex			0.012
Female	32 (50.8)	11 (26.2)	
Male	31 (49.2)	31 (73.8)	
Education			0.352
Under diploma	43 (68.3)	34 (80.9)	
Diploma	12 (19.0)	5 (11.9)	
Academic	8 (12.7)	3 (7.2)	
Occupation			0.033
Employee	4 (6.3)	2 (4.8)	
Self-employed	13 (20.6)	18 (42.8)	
Housekeeper	31 (49.2)	10 (23.8)	
Retired	15 (23.9)	12 (28.6)	
Smoking			0.469
Never	49 (77.8)	32 (76.2)	
Cessation	14 (22.2)	9 (21.4)	
Current	0	1 (2.4)	
Hypertension	34 (54)	19 (45.2)	0.381
Diabetes Mellitus	21 (33.3)	9 (21.4)	0.186
Hyperlipidemia	32 (50.8)	15 (35.7)	0.128
Previous MI	16 (25.4)	8 (19.0)	0.448

HBCR, Hospital-based cardiac rehabilitation; HCR, Hybrid cardiac rehabilitation; MI, Myocardial infarction

* Data are presented as mean±SD or n (%).

**Table 2:** Distinct effects of each program on the psychological symptoms of the study population*

	HBCR				HCR			
	T_1_	T_2_	Paired *t*	P	T_1_	T_2_	Paired* t*	P
								
Depression	5.194.36	3.573.24	3.36	<0.001	4.07±3.06	3.713.29	0.82	0.419
Anxiety	4.273.26	3.062.54	3.58	<0.001	4.50±3.64	3.402.72	2.53	0.015
Stress	9.445.25	7.784.44	3.28	0.002	9.57±4.51	7.644.42	3.12	0.003

HBCR, Hospital-based cardiac rehabilitation; HCR, Hybrid cardiac rehabilitation; T_1_, Pre-intervention scores; T_2_, Post-intervention scores

* Data are presented as mean±SD.

**Table 3:** Comparisons of the HBCR and HCR programs in terms of impact on stress and anxiety

	F (ANCOVA)	P	Eta squared
Anxiety	0.170	0.681	0.002
Stress	0.267	0.607	0.004

HBCR, Hospital-based cardiac rehabilitation; HCR, Hybrid cardiac rehabilitation; ANCOVA, Analysis of covariance

## Discussion

In the present study, we endeavored to assess the effects of HBCR and HCR on the psychological symptoms of patients following CABG. Our results showed that both programs were equally effective in lessening stress and anxiety, while only HBCR was effective in reducing depressive symptoms. This finding is not concordant with the results of a study by Najafi and Nalini.^7^

Our results indicated the efficacy of HBCR, by comparison with HCR, in alleviating depressive symptoms. Depression is one of the most important psychological and health-related diseases. Indeed, it is considered one of the most important risk factors for cardiovascular disease in various epidemiological studies.^13^^-^^15^ The onset of depression may be a consequence of acute cardiac events. Between 15% and 30% of cardiac patients are affected by a depressive disorder, which can limit their physical ability and quality of life and induce a severe psychosocial disability. Depressive disorders can diminish compliance to treatment and as such degrade the efficacy of CR.^1^^, ^^2^ Nonetheless, continuous and active HBCR programs (26 sessions) seem not only to enhance physical functioning and quality of life^   16 ^ but also to successfully control cardiac symptoms such as angina and morbidity^        17 ^ and to boost treatment compliance in comparison with the limited sessions of HCR.^   1 ^

According to the cognitive model, specific cognitive distortions can lead to depression. These cognitive distortions are associated with altered perceptions of internal and external events and are based on early life experiences. The cognitive model suggests that depressed individuals distort environmental inputs according to their inner negative schemas; consequently, the non-concordant information to the dominant cognitive organization is distorted or excluded from consciousness.^18^ Effecting changes to these cognitive structures is a time-consuming process, which is what HBCR programs typically provide. 

In contrast to a previous study by Najafi and Nalini,^7^ our results showed that both programs were equally effective in reducing stress and anxiety levels. Anxiety is defined as a prediction of future threats, and it is associated with muscle tension, vigilance, and avoidance behaviors.^   19 ^ Anxiety usually leads to poor memory and concentration as well as confusion, and it also impairs the individual’s perception of the importance of events such as cardiac attack or surgery. The impaired cognitive function can cause difficulties in perceiving past events correctly. Anxious individuals are liable to report thoughts and emotions of danger and fear during actual situation.^18^ It seems that anxiety is a perceptional response against the non-perfect perceptions among individuals affected by it.^20^ In cardiac patients, anxiety is usually affected by their attitudes toward the future outcomes of the disease. These patients experience more anxiety if they feel a lower sense of control on their disease. Thus, these patients try to enhance their sense of personal control through an active participation in the CR program.^   2 ^ On the other hand, not only do individuals affected by stress tend to feel fear, depression, and anger but also they are likely to express avoidant behaviors and induce reactions in others, which lead to maladaptive stress responses.^18^ Be that as it may, the severity and the quality of stress depend on present challenges and the individual’s perceptions of the disease. As was evidenced by our results, however, both HBCR and HCR programs seemed to be equally effective in increasing adaptive coping strategies in our cardiac patients with respect to their own disease. 

The most limiting aspect of the current study is its nonrandom assignment of the patients to the 2 programs. The study population was assigned to 1 of the 2 groups based on their cardiac risk level, distance from the hospital, and place of residence. Future studies should assess the efficacy of both programs using a randomized controlled trial. Another limitation is that the groups were unbalanced in terms of some characteristics such as sex. The limited number of patients under CR precluded us from performing matching for the groups. That we failed to control the 2 groups apropos their depression scores at baseline is another drawback. What constitute other weaknesses of note are the assessment of the study population’s psychological dimensions through only a questionnaire and the lack of follow-up. Accordingly, we suggest that future studies incorporate clinical interviews, follow-ups, and control of the confounding effects of cardiac drugs such as beta-blockers. 

## Conclusion

Alternative CR programs are not well-known in low-medium-income countries such as Iran. However, the results of the present study showed the efficacy of HCR in alleviating stress and anxiety levels among cardiac patients. Given the cost-effectiveness of HCR compared with HBCR, it is recommended that further studies be conducted on the impact of alternative delivery formats.

## Notes:

***This paper should be cited as:*** Saeidi M, Soroush A, Komasi S, Brugnera A, Patucelli M, Carrozzino D, Fulcheri M, Compare A. Efficacy of Alternative Cardiac Rehabilitation Delivery Formats in Improving Psychological Symptoms after Coronary Artery Bypass Grafting. J Teh Univ Heart Ctr 2018;13(3):103-107.
